# How does angiotensin AT_2_ receptor activation help neuronal differentiation and improve neuronal pathological situations?

**DOI:** 10.3389/fendo.2012.00164

**Published:** 2012-12-19

**Authors:** Marie-Odile Guimond, Nicole Gallo-Payet

**Affiliations:** Division of Endocrinology, Department of Medicine, Faculté de Médecine et des Sciences de la Santé, Université de SherbrookeSherbrooke, QC, Canada

**Keywords:** AT_2_ receptor, angiotensin, brain, differentiation, regeneration, neurodegenerative disorders, signaling, cognitive functions

## Abstract

The angiotensin type 2 (AT_2_) receptor of angiotensin II has long been thought to be limited to few tissues, with the primary effect of counteracting the angiotensin type 1 (AT_1_) receptor. Functional studies in neuronal cells have demonstrated AT_2_ receptor capability to modulate neuronal excitability, neurite elongation, and neuronal migration, suggesting that it may be an important regulator of brain functions. The observation that the AT_2_ receptor was expressed in brain areas implicated in learning and memory led to the hypothesis that it may also be implicated in cognitive functions. However, linking signaling pathways to physiological effects has always proven challenging since information relative to its physiological functions has mainly emerged from indirect observations, either from the blockade of the AT_1_ receptor or through the use of transgenic animals. From a mechanistic standpoint, the main intracellular pathways linked to AT_2_ receptor stimulation include modulation of phosphorylation by activation of kinases and phosphatases or the production of nitric oxide and cGMP, some of which are associated with the Gi-coupling protein. The receptor can also interact with other receptors, either G protein-coupled such as bradykinin, or growth factor receptors such as nerve growth factor or platelet-derived growth factor receptors. More recently, new advances have also led to identification of various partner proteins, thus providing new insights into this receptor’s mechanism of action. This review summarizes the recent advances regarding the signaling pathways induced by the AT_2_ receptor in neuronal cells, and discussed the potential therapeutic relevance of central actions of this enigmatic receptor. In particular, we highlight the possibility that selective AT_2_ receptor activation by non-peptide and selective agonists could represent new pharmacological tools that may help to improve impaired cognitive performance in Alzheimer’s disease and other neurological cognitive disorders.

## INTRODUCTION

It is now well accepted that the effects of the various components of the renin-angiotensin system (RAS) range in various aspects of peripheral and brain functions well beyond those of regulating blood pressure and hydro-mineral balance. In particular, the existence of a complete RAS in the brain is fully acknowledged. Its activation leads to angiotensin II (Ang II) production, which is usually viewed as the end-product of this system (de Gasparo et al., 2000). Ang II binds two receptors from the G protein-coupled receptor family (GPCR), namely the angiotensin type 1 (AT_1_) and angiotensin type 2 (AT_2_) receptor. Although physiological functions of the AT_1_ receptor are relatively well-established, ranging from vasoconstriction and aldosterone release to cell growth, the effects associated with the AT_2_ receptor are surrounded by controversy. Both AT_1_ and AT_2_ receptors are expressed in various brain areas involved in the regulation of fluid and electrolyte balance and in the regulation of arterial pressure, as well as in structures involved in cognition, behavior, and locomotion (Phillips and de Oliveira, 2008; Horiuchi et al., 2010; Horiuchi and Mogi, 2011; Wright and Harding, 2011, 2012; Mogi and Horiuchi, 2012).

One of the biggest challenges in studying the AT_2_ receptor is to apply what has been observed using cell lines to *in vivo* models. Indeed, studies using cell lines expressing the AT_2_ receptor either endogenously or by transfection, have provided paramount information regarding its intracellular mechanisms of action, although associating these mechanisms with biological functions has proven to be much more difficult. Indeed, most of the relevant information regarding AT_2_ receptor functions in the brain has emerged from indirect observations, either by use of AT_1_ receptor blockers (ARB) or *via* transgenic “knock-down” animals for AT_2_ receptor expression. The present review summarizes recent advances in AT_2_ receptor signaling pathways, and discusses how they could be related to the neuroprotective functions of the receptor.

## BRAIN EXPRESSION AND ROLE OF THE AT_2_ RECEPTOR

As summarized in several reviews (de Gasparo et al., 2000; Porrello et al., 2009; Gallo-Payet et al., 2011; Wright and Harding, 2011; Mogi and Horiuchi, 2012), the AT_2_ receptor is widely expressed during fetal life, which decreases rapidly after birth (Grady et al., 1991; Breault et al., 1996; Schutz et al., 1996; Nuyt et al., 1999), although a recent study has reported opposite results (Yu et al., 2010). This study is indeed in sharp contrast with previous reports using more specific methods, like autoradiography or *in situ* hybridization. In the adult, AT_2_ receptor expression is limited to a few tissues and cell types, such as vascular endothelial cells, adrenal gland, kidney, heart, myometrial cells, and ovaries (review in Porrello et al., 2009; Gallo-Payet et al., 2011, 2012; Verdonk et al., 2012). In the adult central nervous system (CNS), the AT_2_ receptor is observed in certain specific brain areas involved in the control and learning of motor activity, control of autonomous functions, sensory areas, and selected limbic system structures (Lenkei et al., 1996, 1997). In particular, it is the major Ang II receptor in the medulla oblongata (control of autonomous functions), septum and amygdala (associated with anxiety-like behavior), thalamus (sensory perception), superior colliculus (control of eye movements in response to visual information) as well as subthalamic nucleus and cerebellum (areas associated with learning of motor functions). On the other hand, certain areas involved in cardiovascular functions, learning, behavior, and stress reactions (cingulate cortex, molecular layer of the cerebellar cortex, superior colliculus, and paraventricular nuclei) contain both AT_1_ and AT_2_ receptors (Millan et al., 1991; Tsutsumi and Saavedra, 1991; Lenkei et al., 1996, 1997). More recently, expression of the AT_2_ receptor was also detected in the substantia nigra pars compacta, an area involved in dopaminergic signals and associated with Parkinson’s disease (Grammatopoulos et al., 2007), and in the hippocampus (Arganaraz et al., 2008; AbdAlla et al., 2009). At the cellular level, the AT_2_ receptor is expressed in neurons, but not in astrocytes (Bottari et al., 1992a; Lenkei et al., 1996; Gendron et al., 2003). Evidence also suggests that the AT_2_ receptor is expressed in the vasculature wall, where it acts on cerebral blood flow (review in Horiuchi and Mogi, 2011; Horiuchi et al., 2012). It should also be noted that existence of a non-AT_1_/non-AT_2_ receptor in the CNS has been suggested, which displays high affinity for Ang I, II, and III (Karamyan and Speth, 2007).

### ROLE OF THE AT_2_ RECEPTOR IN NEURONAL EXCITABILITY

One of the first roles of the AT_2_ receptor to be identified was the modulation of neuronal excitability, which plays a crucial role not only in neuronal differentiation, but also in neuronal functions (review in Gendron et al., 2003; Gao et al., 2011). In particular, in cells of neuronal origin, activation of the AT_2_ receptor decreases activity of T-type calcium channels (Buisson et al., 1992, 1995). On the other hand, in rat brain neuronal culture, Kang et al. (1994) showed that the AT_2_ receptor stimulates a delayed rectifier K^+^ current (I_K_) and a transient K^+^ current (I_A_), an effect dependent on the G-protein Gi and the serine/threonine phosphatase PP2A. Consistent with these observations, a recent study showed that AT_2_ receptor induces a hyperpolarization and a decrease in firing rate in rostral ventrolateral medulla (RVLM) neurons suggesting that central activation of the AT_2_ receptor in this region decreases excitability (Matsuura et al., 2005). More recently, another study using C21/M024 demonstrated that selective stimulation of AT_2_ receptor in the neuronal cell line (called CATH.a neurons) increases the potassium current activity (*I*_Kv_)_(Kv)_ in a nitric oxide (NO)-dependant pathway (Gao et al., 2011). Moreover, intracerebroventricular infusion of C21/M024 was associated with a decrease in norepinephrine excretion and in blood pressure. Indeed, the modulation of the receptor on neuronal excitability in this region could be one of the mechanism associated with its effect on blood pressure, since RVLM is often considered as the main regulator of vascular tone (review in Dupont and Brouwers, 2010). An inhibitory effect of the AT_2_ receptor on neuronal excitability has also been observed in the locus coeruleus from brain slice preparations (Xiong and Marshall, 1994) and in the superior colliculus (Merabet et al., 1997). Finally, using the selective agonist C21/M024, Jing et al. (2012) recently demonstrated that direct stimulation of cerebral AT_2_ receptor increases postsynaptic potential, thus corroborating previous *in vitro* observations. Interestingly, AT_2_ receptor-induced neuronal activation of delayed rectifier potassium channels has also been demonstrated to have a neuroprotective effect (Grammatopoulos et al., 2004a). In fact, these AT_2_ receptor effects on ionic channel activity suggest that it may be implicated in synaptic plasticity, an important process involved in learning and memory.

### ROLE OF THE AT_2_ RECEPTOR IN NEURONAL DIFFERENTIATION

One of the best recognized effects of AT_2_ receptor stimulation in neuronal cells is the induction of neurite outgrowth (review in Gallo-Payet et al., 2011). In the early 1990s, our group observed that stimulation of the AT_2_ receptor with its selective agonist CGP42112A induces neurite outgrowth in the neuronal NG108-15 cell line (Laflamme et al., 1996), results that were further confirmed using the recently developed non-peptide selective AT_2_ receptor agonist C21/M024 (Wan et al., 2004). This effect was associated with an increase in mature neural cell markers, such as βIII-tubulin, and microtubule-associated proteins (MAPs) such as MAP2c (Laflamme et al., 1996), both known to stabilize tubulin in a polymerized state, thus participating actively in differentiation (Sanchez et al., 2000). Similar results have also been reported in the pheochromocytoma-derived cell line PC12W, where Ang II was found to promoted neuronal differentiation characterized by an increase in neurite elongation (Meffert et al., 1996) and enhanced levels of polymerized βIII-tubulin and MAP2 associated with microtubules (Stroth et al., 1998). However, neurite outgrowth in PC12W cells has also been associated with a reduced expression of MAP1B (Stroth et al., 1998) and neurofilament M (Gallinat et al., 1997), two proteins specifically associated with axon elongation (Gordon-Weeks, 1991). These results were further confirmed in primary neuronal cultures, including retinal explants (Lucius et al., 1998), microexplant cultures of the cerebellum (Coté et al., 1999), in neurospheres from mouse fetal brain (Mogi et al., 2006) as well as primary cultures of newborn brain cortex neurons (Li et al., 2007) and hippocampal neurons (Jing et al., 2012). Some studies also showed that this neurite elongation was associated with an increase in the repair of damaged DNA by induction of methyl methanesulfonate sensitive-2 (MMS2), a neural-differentiating factor (Mogi et al., 2006; Jing et al., 2012). Altogether, these results suggest that activation of the AT_2_ receptor is associated with important rearrangements of the cytoskeleton necessary for induction of neurite elongation.

### ROLE OF THE AT_2_ RECEPTOR IN NEURONAL MIGRATION

In cerebellar microexplants, where both neuronal and glial cells are present, AT_2_ receptor activation induces not only neurite outgrowth, but cell migration as well (Coté et al., 1999). Indeed, application of Ang II in this model induced cell migration of neurons from the center toward the periphery of the microexplant (Coté et al., 1999). These effects were more pronounced in cells treated with Ang II and DUP 753 (known as the ARB losartan) or in cells treated with 10 nM of CGP42112A an AT_2_ receptor agonist, and conversely blocked with the AT_2_ receptor antagonist PD123,319. Similar cell migration has also been observed during AT_2_ receptor-induced regeneration of post-natal retinal microexplants (Lucius et al., 1998). During migration and neurite outgrowth, cells are characterized by a myriad of advancing, retracting, turning, and branching behavioral patterns. Such dynamics and plasticity are driven by the reorganization of actin and the microtubular cytoskeleton. In particular, during the process of migration, actin filaments play a major role and are putatively considered as the primary target of guidance cues, due to their localization at the cell periphery, and in filopodium in the growth cone, where they are considered to be the driving force for the forward extension of the cell membrane (Gallo and Letourneau, 2004; Kalil and Dent, 2005). Our results on NG108-15 cells have shown that the underlying mechanism involves an Ang II-induced decrease in the amount of F-actin in filopodium and an increase in the pool of unpolymerized actin, through a pertussis toxin (PTX)-sensitive increase in ADF/cofilin activity. These latter effects were found to be AT_2_ receptor-dependent, since the increase in the rate of migration was abolished by the selective antagonist PD123,319, but not by the selective AT_1_ receptor antagonist losartan. Interestingly, some co-localization of F-actin with microtubules was also observed in control conditions, but which disappeared during Ang II-induced migration (Kilian et al., 2008). Among the candidate molecules that possibly cross-link actin filaments and microtubules are MAP2c and MAP1B (Dehmelt et al., 2003; Dehmelt and Halpain, 2004), proteins previously shown by our group to be affected during the process of AT_2_ receptor-stimulated neurite outgrowth, both in NG108-15 cells and in cerebellar granule cells (Laflamme et al., 1996; Coté et al., 1999).

## MAIN SIGNALING PATHWAYS OF THE AT_2_ RECEPTOR

**FIGURE 1:**
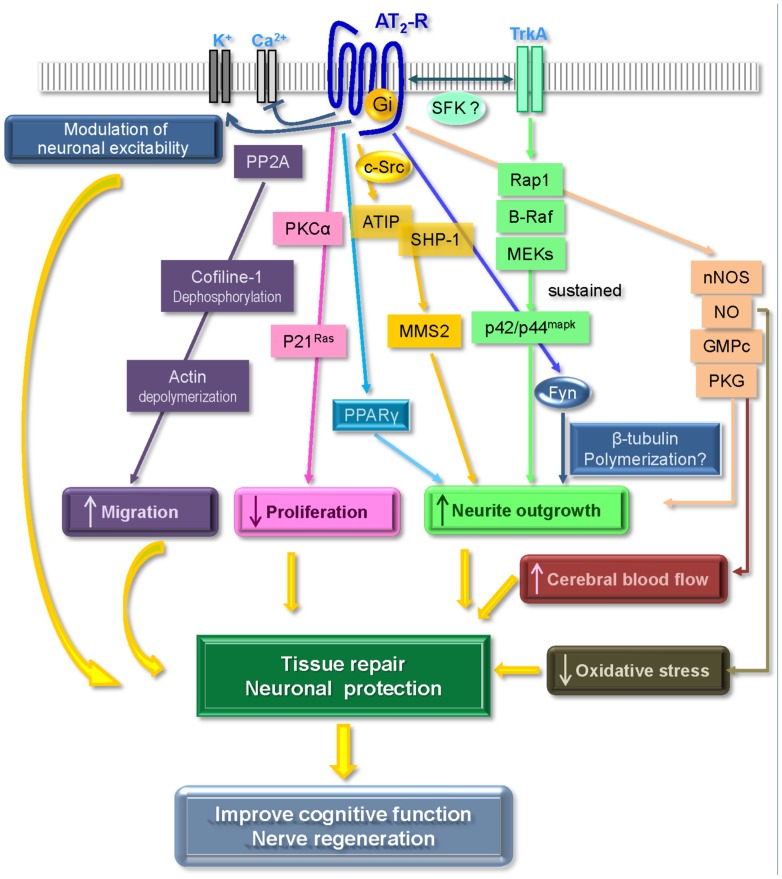
**Main signaling pathways associated with AT_2_ receptor activation leading to neuroprotective effects (see text for details).** Adapted from Gallo-Payet et al. (2011).

Although the AT_2_ receptor displays most of the classical features of a GPCR, it is usually considered as an atypical member of this family, since it fails to induce all of the classical signaling pathways such as cAMP, production of inositol triphosphate (IP3) or intracellular calcium release. Signaling pathways associated with the AT_2_ receptor mainly involve a balance between phosphatase and kinase activities and according to whether the cell is undifferentiated or differentiated and whether it expresses angiotensin AT_1_ receptors or not. Thus, there is still much controversy surrounding this receptor, and its effects, either protective or deleterious, remain a subject of debate (Widdop et al., 2003; Steckelings et al., 2005, 2010; Porrello et al., 2009; Horiuchi et al., 2012; Verdonk et al., 2012). In our endeavor to elucidate the mechanisms associated with AT_2_ receptor-induced neurite outgrowth, we and others have investigated signaling pathways activated by this receptor, including G-protein coupling, regulation of kinase activity, interaction with growth factor receptors, and production of NO. Moreover, recent observations have also delineated new partners for the AT_2_ receptor which play key functions in its regulation (**Figure 1**).

### G-PROTEIN COUPLING

While coupling of G-protein to AT_1_ receptors is well described (de Gasparo et al., 2000; Hunyady and Catt, 2006), such coupling is not the rule for the AT_2_ receptor. Former studies have described a coupling to subunit Gα_i__2_ and Gα_i__3_ in rat fetus (Zhang and Pratt, 1996). In some models (rat hippocampal neurons and other selected cell types), blocking Gα_i_ with PTX or antibodies directed against Gα_i_ inhibited the AT_2_ receptor effects on actin depolymerization, activation of endothelial NO synthase (NOS), stimulation of neuronal K^+^ current and on anti-proliferative activity (Kang et al., 1994; Ozawa et al., 1996; Li et al., 2004; Olson et al., 2004; Kilian et al., 2008), indicating that coupling of the AT_2_ receptor to Gα_i_ is at least implicated in these pathways. However, aside from a few exceptions (Kang et al., 1994), PTX failed to inhibit either p42/p44^mapk^ activation in the neuronal cell line NG108-15 (Gendron et al., 2002) or phosphatase activity in several models (for review see Nouet and Nahmias, 2000; Gendron et al., 2003).

### REGULATION OF KINASE ACTIVITY

#### AT_2_ Receptor-induced phosphatase activation

Phosphatase activation has been one of the first signals associated with AT_2_ receptor activation. After the earlier studies in PC12W cells (Bottari et al., 1992b; Brechler et al., 1994), results have been confirmed in other cell lines, including N1E-115 cells (Nahmias et al., 1995), NG108-15 cells (Buisson et al., 1995), and R3T3 fibroblasts (Tsuzuki et al., 1996a,b). This phosphatase activation by the AT_2_ receptor is essential for its anti-proliferative and pro-apoptotic effects (for reviews, see Nouet and Nahmias, 2000; Steckelings et al., 2005; Porrello et al., 2009; Verdonk et al., 2012). Currently, three main phosphatases have been implicated in AT_2_ receptor signaling, namely SH2-domain-containing phosphatase 1 (SHP-1), mitogen-activated protein kinase phosphatase 1 (MKP-1), and the serine–threonine phosphatase PP2A.

SHP-1 is a cytosolic phosphatase rapidly activated by the AT_2_ receptor following Ang II binding. Activation of SHP-1 is associated with AT_2_-induced growth inhibition in various cells, including neuronal cells (Bedecs et al., 1997; Elbaz et al., 2000; Feng et al., 2002; Li et al., 2007), vascular smooth muscle cells (Cui et al., 2001; Matsubara et al., 2001), CHO, and COS-7 cells transfected with the AT_2_ receptor (Elbaz et al., 2000; Feng et al., 2002). Activation of SHP-1 is associated with inhibitory effects of the AT_2_ receptor on the AT_1_ receptor, including transactivation of the epidermal growth factor (EGF) receptor and activation of c-Jun N-terminal kinase (JNK) (Matsubara et al., 2001; Shibasaki et al., 2001), but also on insulin-induced activation of the phosphatidylinositol 3-kinase (PI3K), its association with the insulin receptor substrate IRS-2 and phosphorylation of Akt (Cui et al., 2001). This inhibition of insulin signaling by AT_2_ receptor-induced SHP-1 activation has also been associated with an increase in PC12W cell apoptosis (Cui et al., 2002). More recently, Li et al. (2007) have shown that induction of neurite outgrowth in fetal rat neurons by AT_2_ receptor involves the association of SHP-1 with the newly identified AT_2_-receptor interacting protein (ATIP; see section AT_2_ Receptor Interacting Proteins) and an increase in MMS2 protein (Li et al., 2007). Finally, although the mechanisms associated with AT_2_ receptor-induced activation of SHP-1 have yet to be fully elucidated, implication of G-protein coupling (Bedecs et al., 1997; Feng et al., 2002) as well as activation of Src kinase (Alvarez et al., 2008) have been reported; other studies have also implicated a constitutive association between AT_2_ receptor and SHP-1 in overexpressing models (Feng et al., 2002; Miura et al., 2005). Another phosphatase associated with AT_2_ receptor activation is MKP-1, which is a key regulator of p42/p44^mapk^ activity. AT_2_ receptor-activated MKP-1 has been observed in various cell types, including PC12W cells (Yamada et al., 1996), fibroblasts (Horiuchi et al., 1997; Calo et al., 2010), and cardiac myocytes (Fischer et al., 1998; Hiroi et al., 2001). Activation of MKP-1 by AT_2_ leads to a decrease in p42/p44^mapk^ activity, and is associated to growth inhibition induced by the AT_2_ receptor. Moreover, Horiuchi et al. (1997) demonstrated that AT_2_ receptor-induced MKP-1 activation is implicated in apoptotic effects of the AT_2_ receptor, leading to Bcl-2 dephosphorylation and an increase in Bax, resulting in cell death. Finally, the serine–threonine phosphatase PP2A is also activated by the AT_2_ receptor following Ang II binding and may be associated with AT_2_ receptor regulation of p42/p44^mapk^. Indeed, in primary neuronal cultures, AT_2_ receptor-induced activation of PP2A is associated with inhibition of AT_1_ receptor-induced p42/p44^mapk^ phosphorylation (Huang et al., 1995, 1996a,b) and is implicated in AT_2_-induced modulation of potassium currents (Huang et al., 1995, 1996a; Caballero et al., 2004). More recently, we have also shown an implication of PP2A activation in actin depolymerization and an increase in neuronal migration (Kilian et al., 2008; **Figure 1**).

#### Mitogen-activated protein kinase p42/p44

Among all signaling pathways associated with AT_2_ receptor activation, regulation of p42/p44^mapk^ is probably the one where variability is the most important. The effect of AT_2_ receptor stimulation on activation or inhibition of p42/p44^mapk^ activity is dependent on the models studied, on whether they express AT_1_ receptors or not and whether cells are under physiological or pathological conditions. Thus, AT_2_ receptor effects on p42/p44^mapk^ remain controversial. Many studies have shown that the AT_2_ receptor leads to dephosphorylation of p42/p44^mapk^ *via* one the phosphatases associated with AT_2_ receptor signaling (see above). This decrease in p42/p44^mapk^ activity is associated with inhibition of growth and pro-apoptotic effects of the AT_2_ receptor (review in Nouet et al., 2004; Porrello et al., 2009). In addition to activation of phosphatase, AT_2_ receptor-induced inhibition of p42/p44^mapk^ can be mediated by inhibition of growth factor receptors. Indeed, in vascular smooth muscle cells overexpressing the AT_2_ receptor, stimulation with Ang II decreases EGF receptor phosphorylation and inhibits p42/p44^mapk^ activation (Shibasaki et al., 2001). Similar observations have also been reported in CHO cells overexpressing the AT_2_ receptor (Elbaz et al., 2000). Worthy of note is the fact that inhibition of p42/p44^mapk^ induced by the AT_2_ receptor is observed only in certain conditions, such as in cells overexpressing the AT_2_ receptor or already exhibiting pathological conditions such as serum-starving (Bedecs et al., 1997; Horiuchi et al., 1997; Elbaz et al., 2000; Cui et al., 2001; Shibasaki et al., 2001).

By contrast, in neuronal cells such as NG108-15 and PC12W cells, the AT_2_ receptor leads to sustained activation of p42/p44^mapk^. In these cells, activation of p42/p44^mapk^ is essential to AT_2_ receptor-induced neurite elongation (Gendron et al., 1999; Stroth et al., 2000). In NG108-15 cells, we observed that this increase in p42/p44^mapk^ activity was associated with the Rap1/B-Raf pathway. However, this Rap1 activation appears to be dependent of nerve growth factor receptor TrkA activation (see latter; Plouffe et al., 2006) rather than through cAMP and protein kinase A (PKA), as usually observed with other GPCR (**Figure 1**). This activation of p42/p44^mapk^ by the AT_2_ receptor has also been observed in non-neuronal COS-7 and NIH3T3 cells overexpressing the AT_2_ receptor (Hansen et al., 2000; De Paolis et al., 2002).

#### Src family kinase

There are few studies showing an implication of Src family members in AT_2_ receptor signaling. However, Src family kinases (SFKs) are key regulators in cell growth and differentiation and are implicated in most growth factor signaling pathways. In the CNS, five members of SFK are expressed, namely Src, Fyn, Lyn, Lck, and Yes, where they act as modulators of neurotransmitter receptors as well as in the regulation of excitatory transmission (review in Kalia et al., 2004; Theus et al., 2006; Ohnishi et al., 2011). Recently, we have shown that stimulation of the AT_2_ receptor in NG108-15 cells leads to rapid but transient activation of SFK and that expression of inactive Fyn abolished AT_2_ receptor-induced neurite outgrowth in these cells (Guimond et al., 2010). However, inhibition of Fyn had no effect on other signaling pathways induced by the AT_2_ receptor, including p42/p44^mapk^ and Rap1 activation, suggesting that it may be involved either downstream of these proteins, or in a parallel pathway. Of note, among the five SFKs expressed in the brain, only a deficiency in Fyn-induced neurological deficits, including impairment in spatial learning and in hippocampal development (Grant et al., 1992; Kojima et al., 1997). Interestingly, similar physiological perturbations were also observed in mice lacking the AT_2_ receptor (Hein et al., 1995; Ichiki et al., 1995; Okuyama et al., 1999; Maul et al., 2008). Therefore, regulation of Fyn activity could be considered as a new player implicated in the protective effect of this receptor in cognitive disorders. Indeed, Fyn has been shown to be involved in tau phosphorylation, thus regulating its affinity for tubulin and stability of microtubules, two parameters implicated in the development of Alzheimer’s disease (AD) and other neurodegenerative diseases (Lee et al., 1998, 2004). Thus, it appears that Fyn is involved in the final steps of induction of elongation, but not in the initial events of AT_2_ receptor activation. This implication of Fyn in AT_2_ receptor signaling is further strengthened by the fact that activation of SFKs, as the AT_2_ receptor, was shown to be important for the induction of long-term potentiation, a key element in learning and memory, in CA1 pyramidal neurons of hippocampal slices (Yu et al., 1997).

To the best of our knowledge, only one other group has demonstrated the implication of a Src family member in AT_2_ receptor signaling (Alvarez et al., 2008). In this latter study, it was shown that activation of c-Src was present in an immunocomplex including the tyrosine phosphatase SHP-1 and the AT_2_ receptor following Ang II stimulation in rat fetal membranes. Pre-incubation of membranes with the non-selective inhibitor PP2 inhibited SHP-1 activation and c-Src association. These results indicate that c-Src may represent an important step leading to AT_2_ receptor-induced SHP-1 activation. More recently, the same group demonstrated that this association also occurred in hindbrain membranes from post-natal day 15 rats, and was associated with focal adhesion kinase (p85FAK) (Seguin et al., 2012). These observations strongly suggest that c-Src may also be implicated in cytoskeleton remodeling associated with neurite elongation and neuronal migration induced by the AT_2_ receptor.

### LINKING THE AT_2_ RECEPTOR WITH THE GROWTH FACTOR RECEPTORS

Recently, we demonstrated that activation of Rap1/B-Raf/p42/p44^mapk^ pathway by the AT_2_ receptor was dependent on the nerve growth factor receptor TrkA, although the mechanism involved remains unknown (Plouffe et al., 2006). In addition, we further showed that a SFK member was essential for the initial activation of TrkA by the AT_2_ receptor, since pre-incubation of NG108-15 cells with the non-selective inhibitor PP1 disrupted this effect (Guimond et al., 2010). However, although Fyn was essential for neurite outgrowth induced by the AT_2_ receptor, it did not appear to be implicated in TrkA activation, since expression of a dominant negative form did not impede AT_2_-induced TrkA activation (Guimond et al., 2010). In light of recent data obtained by Ciuffo’s group regarding the involvement of c-Src and other SFK members with AT_2_ receptors (Alvarez et al., 2008; Seguin et al., 2012), it would be of interest to see whether the association of the AT_2_ receptor with SHP-1 and c-Src is implicated in this transactivation, and whether TrkA could be involved in FAK activation. Interestingly, transactivation of the TrkA receptor in neurons has also been observed for the pituitary adenylyl cyclase-activating polypeptide receptor (PACAP; Rajagopal et al., 2004), which is also associated with neuronal development in the cerebellum (Basille et al., 2006).

Curiously, although the expression of inactive Fyn is known to disrupt AT_2_ receptor-induced neurite elongation, non-selective inhibition of SFK in NG108-15 cells with the inhibitor PP1 is sufficient to increase neurite elongation to levels similar to those observed with AT_2_ receptor stimulation (Guimond et al., 2010), which could be a consequence of a decrease in proliferative signal. Indeed, our group showed that induction of neurite outgrowth was associated with a decrease in cell proliferation through inhibition of PKCα and p21^Ras^ (Gendron et al., 1999; Beaudry et al., 2006). Moreover, as in the case of SFK, inhibition of the platelet-derived growth factor (PDGF) receptor was sufficient to induce neurite outgrowth and to increase microtubule polymerization more extensively than Ang II alone (Plouffe et al., 2006). These findings are in agreement with a previous report demonstrating that expression of an inactive form of the PDGF receptor in PC12 cells was sufficient to increase neurite elongation (Vetter and Bishop, 1995). However, whether AT_2_ receptor directly inhibits PDGF receptor or inhibits its signaling pathway is still unknown.

### NITRIC OXIDE AND CGMP PRODUCTION – A ROLE FOR BRADYKININ

Nitric oxide has been shown to regulate several types of K^+^ channels, including ATP-dependent K^+^ channels and Ca^2^^+^-activated K^+^ channels (review in Prast and Philippu, 2001). Indeed, in neuronal cell lines, observations with the selective AT_2_ receptor agonist C21/M024 revealed that this production of NO induced by AT_2_ was necessary for AT_2_-induced hyperpolarization of potassium channel function (Gao and Zucker, 2011). Production of NO following AT_2_ receptor stimulation has been observed in various cell types, such as neuronal cells (Chaki and Inagami, 1993; Coté et al., 1998; Gendron et al., 2002; Zhao et al., 2003; Muller et al., 2010), vascular endothelial cells (Wiemer et al., 1993; Seyedi et al., 1995; Saito et al., 1996; Thorup et al., 1998; Baranov and Armstead, 2005) as well as in smooth muscle cells (de Godoy et al., 2004). It is already well accepted that AT_2_ receptor activation plays an important role in the control of renal function particularly in chronic kidney diseases. The AT_2_ receptor is believed to counterbalance the effects of the AT_1_ receptor at least by influencing vasodilation through NO production and natriuresis (Carey and Padia, 2008; Siragy, 2010; Siragy and Carey, 2010). This promoter effect of AT_2_ on natriuresis in pathological conditions (obese Zucker rats) was also recently confirmed using C21/M024 (Ali and Hussain, 2012). Activation of NOS by the AT_2_ receptor can occur by direct signaling such as in neuronal cells, or indirectly *via* stimulation of bradykinin production and subsequent activation of its receptor B2. Indeed, heterodimerization between the AT_2_ receptor and bradykinin has also been described in PC12W cells (Abadir et al., 2006). Moreover, it is already known that bradykinin can modulate AT_2_ receptor-induced NO production (Siragy and Carey, 1996; Gohlke et al., 1998; Searles and Harrison, 1999). Such involvement of B2 receptors in AT_2_ receptor-induced production of NO is of prime importance in the modulation of cerebral blood flow. Indeed, an AT_2_-induced increase in spatial learning was recently observed to be associated with an increase in cerebral blood flow, an effect reduced by co-administration of the B2 receptor antagonist icatibant. This observation strongly suggests that the beneficial effect of the AT_2_ receptor in cognitive function is partly dependent on bradykinin (Jing et al., 2012). In addition, Abadir et al. (2003) demonstrated in conscious bradykinin B2-null and wild-type mice that the AT_2_ receptor can induce production of NO in both null and wild-type models, indicating that the B2 receptor may participate in this process, although is not the only means for the AT_2_ receptor to induce NO production.

### AT_2_ RECEPTOR ASSOCIATED PROTEINS

#### ATIP

Recently, using a yeast two-hybrid system, the ATIP was cloned and identified as a protein interacting with the C-terminal tail of the AT_2_ receptor (Nouet et al., 2004). This protein is expressed as five different transcripts, namely ATIP1, ATIP2, ATIP3a, ATIP3b, and ATIP4 (review in Rodrigues-Ferreira and Nahmias, 2010; Horiuchi et al., 2012). While ATIP3 appears to be the major transcript in tissues, ATIP1 and ATIP4 are mainly expressed in the brain, indicating that they may play biological roles in brain functions. ATIP2, on the other hand, is almost undetectable by real-time PCR (Di Benedetto et al., 2006). In CHO cells expressing the AT_2_ receptor, ATIP is known to decrease growth factor-induced p42/p44^mapk^ activation and DNA synthesis, therefore decreasing cell proliferation, as well as decrease insulin receptor autophosphorylation, similarly to the AT_2_ receptor. Of particular interest is the fact that, although expression of the AT_2_ receptor was essential in this instance, stimulation by Ang II was not necessary, and that ATIP was able to exert its effect by its sole expression. Implication of ATIP in AT_2_ receptor-induced neurite outgrowth has also been reported. In this context, Ang II stimulation of the AT_2_ receptor induces translocation of ATIP with SHP-1 into the nucleus, resulting in the transactivation of MMS2 (Li et al., 2007). Moreover, ATIP, also known as ATBP50 (AT_2_ receptor binding protein of 50 kDa), has been reported as a membrane-associated Golgi protein implicated in intracellular localization of the AT_2_ receptor and necessary for its membrane expression (Wruck et al., 2005). ATIP3, which is also expressed in the CNS, has been shown to strongly interact with stabilized microtubules in a model of breast cancer, suggesting an implication on cell division, where it induces a delayed metaphase, thus decreasing tumor progression (Rodrigues-Ferreira et al., 2009). The brain-specific isoform ATIP4 is highly expressed in the cerebellum and fetal brain, two sites where the AT_2_ receptor is also highly expressed. Therefore considering (i) the previously described function of the AT_2_ receptor in preservation of cognitive function, (ii) the role of ATIP protein in AT_2_ receptor function, and (iii) the link between ATIP protein and microtubule cytoskeleton, it could be suggested that regulation of ATIP expression and regulation of its association with the AT_2_ receptor could be an important element to consider with regard to the development of neurological disorders, such as AD.

#### PLZF

Association between the AT_2_ receptor and the promyelocytic leukemia zinc finger (PLZF) protein has been observed using a yeast two-hybrid system (Senbonmatsu et al., 2003). In CHO cells expressing both PLZF and AT_2_ receptors, Ang II stimulation induces co-localization of PLZF with the AT_2_ receptor, followed by internalization of the complex. This observation is in contrast with other studies observing no internalization of the AT_2_ receptor following Ang II stimulation (Hunyady et al., 1994; Hein et al., 1997). Since internalization of the receptor was observed only in cells expressing PLZF, this could represent a new regulatory pathway of AT_2_ receptor function, specific only to selected cell types. However, beside internalization of AT_2_ receptor, a recent study showed that PLZF was implicated in neuroprotection in a stroke model (Seidel et al., 2011). In this study, the authors showed that PLZF exerts neuroprotective effect in a model of *in vitro* glutamate toxicity. They also showed that overexpression of PLZF in neuronal cells in culture induced a significant increase in AT_2_ receptor expression, suggesting that PLZF could also be implicated in the regulation of AT_2_ receptor expression.

#### PPARγ

A new partner for the AT_2_ receptor has recently emerged from the study of Zhao et al. (2005) who observed that neurite outgrowth induced by AT_2_ receptor stimulation in PC12W cells was dependent on the activation of peroxisome proliferator-activated receptor gamma (PPARγ). This observation is in keeping with the implication of PPARγ in NGF-induced neurite outgrowth in the same cell type (Fuenzalida et al., 2005), clearly suggesting a possible crosstalk between the AT_2_ receptor and NGF pathways. This hypothesis is further reinforced by the observation that inhibition of the NGF receptor TrkA significantly decreases AT_2_ receptor-induced neurite outgrowth (Plouffe et al., 2006). Moreover, Iwai et al. (2009), using atherosclerotic ApoE-KO mice with an AT_2_ receptor deficiency (AT2R/ApoE double knockout mice), observed that the lack of AT_2_ receptor expression decreased the expression of PPARγ in adipocytes cells. These observations strongly suggest a link between the AT_2_ receptor and PPARγ functions. PPARγ is a transcriptional factor regulating the expression of multiple genes, hence promoting the differentiation and development of various tissues, specifically in adipose tissue, brain, placenta, and skin. Interestingly, neuroprotective effects of PPARγ agonist have also been observed (review in Gillespie et al., 2011). However, a major component of the hypothesis regarding the possible implication of PPARγ in AT_2_ receptor function is the PPARγ-like activity associated with certain ARBs, including telmisartan, irbesartan, and candesartan (Benson et al., 2004; Schupp et al., 2004; review in Horiuchi et al., 2012). Indeed, there is some evidence suggesting that this PPARγ activation following blockade of the AT_1_ receptor could be part of its anti-inflammatory and anti-oxidative effects, leading to neuroprotection against ischemia and amyloid β (Aβ) accumulation (Tsukuda et al., 2009; Iwanami et al., 2010; Washida et al., 2010). PPARγ has also been implicated in neural cell differentiation and death, as well as inflammatory and neurodegenerative conditions (review in Gillespie et al., 2011).

## LESSONS FROM NEURONAL DIFFERENTIATION: HOW CAN THE AT_2_ RECEPTOR IMPROVE BRAIN FUNCTION?

### ROLE OF THE AT_2_ RECEPTOR IN NEURONAL REGENERATION

The capacity for nerve regeneration in lower vertebrates has been mostly lost in higher vertebrates and regeneration within the CNS in mammals is essentially inexistent. However, after injury in the peripheral nervous system, regeneration can be achieved successfully. Observations that AT_2_ receptor stimulation induces neurite elongation associated with modulation of MAP expression strongly suggested that this effect could also be observed following nerve injury. In 1998, two studies demonstrated that the AT_2_ receptor improved nerve recovery in both optic (Lucius et al., 1998) and sciatic (Gallinat et al., 1998) nerve following nerve crush or in perivascular nerves implicated in vasodilation (Hobara et al., 2007). This effect was accompanied by an increase in AT_2_ receptor expression, the activation of NFκB and induction of growth-associated protein (GAP-43) leading to a reduction in lesion size. Moreover, Reinecke et al. (2003) demonstrated that activation of NFκB by the AT_2_ receptor was an essential step to recovery following sciatic nerve crush. This implication of AT_2_ receptor in neuronal regeneration has even led to the suggestion that Ang II, *via* the AT_2_ receptor, could act as a neurotrophic factor.

### AT_2_ RECEPTOR IN COGNITIVE FUNCTION

There is increasing evidence suggesting that the AT_2_ receptor could be associated with improvement of cognitive function following cerebral ischemia-induced neuronal injury (Iwai et al., 2004; Li et al., 2005; Mogi et al., 2006; McCarthy et al., 2009). Indeed, it has been shown that central administration of CGP42112A increases neuronal survival and minimizes experimental post-stroke injury (McCarthy et al., 2009), indicating that activation of brain AT_2_ receptors exhibits a neuroprotective effect. More recently, stimulation of the AT_2_ receptor with the selective agonist C21/M024 was observed to prevent cognitive decline in an AD mouse model with intracerebroventricular injection of Aβ(1-40) (Jing et al., 2012). Indeed, some of the signaling pathways described above may be linked to improvement in impaired signaling functions as observed in AD. One of the major hallmarks of AD is Aβ deposition in senile plaques and the presence of neurofibrillary tangles (NFTs). Formation of NFTs is a consequence of protein tau accumulation, due to its hyperphosphorylation, and the dissociation of microtubules. Thus, regulation of tau phosphorylation is of paramount importance with regard to AD progression. On the other hand, several studies have reported that the AT_2_ receptor activates PP2A phosphatase (Huang et al., 1995, 1996a; Kilian et al., 2008), which is markedly deficient in AD (Gong et al., 1993, 2000; Wang et al., 2007) and implicated in glycogen synthase kinase-3 (GSK-3) inactivation *via* a sustained increase in p42/p44^mapk^. Since tau is a substrate for PP2A phosphatase, GSK-3 and Fyn, the latter of which is also implicated in the AT_2_ receptor effect on neurite outgrowth (Guimond et al., 2010), AT_2_ receptor activation could participate in controlling the equilibrium between tau phosphorylation and dephosphorylation (Hernandez and Avila, 2008; Hanger et al., 2009; Hernandez et al., 2009). In addition to acting on tau regulation, the AT_2_ receptor may also improve neurite architecture, through effects on MAPs, as observed in neuronal cell lines (Laflamme et al., 1996; Meffert et al., 1996; Coté et al., 1999; Li et al., 2007). The observation that central AT_2_ receptor activation using its selective agonist C21/M024 decreases cognitive loss induced by Aβ intracerebroventricular injection lends further support to this hypothesis (Jing et al., 2012). Although the mechanisms underlying these neuroprotective effects of the AT_2_ receptor remain to be fully elucidated, they may include PPARγ and the protein MMS2 (Mogi et al., 2006, 2008; for recent reviews see Gallo-Payet et al., 2011, 2012).

Moreover, as indicated earlier, another important feature of AT_2_ receptor signaling is induction of NO and cGMP production. Recently, Jing et al. (2012) observed that direct stimulation of central AT_2_ receptors increases NO *via* a bradykinin-dependent pathway, an effect which leads to an increase in cerebral blood flow and enhanced spatial memory. A further study also showed that administration of C21/M024 reduced early renal inflammatory response with production of NO and cGMP (Matavelli et al., 2011). This increase in NO-cGMP production has also been shown to lead to a decrease in nicotinamide adenine dinucleotide phosphate-oxidase (NADPH) superoxide production (Volpe et al., 2003; Widdop et al., 2003; de la Torre, 2004; Steckelings et al., 2005; Iadecola et al., 2009), thus reducing oxidative stress and potentially associated neuronal apoptosis. This hypothesis is coherent with the observation that the AT_2_ receptor attenuates chemical hypoxia-induced caspase-3 activation in primary cortical neuronal cultures (Grammatopoulos et al., 2004b). Finally, inflammation is also a common feature of neurodegenerative diseases. In this regard, a recent study conducted in primary cultures of human and murine dermal fibroblasts, has shown that C21/M024 has anti-inflammatory effects, inhibiting tumor necrosis factor (TNF)-α-induced interleukin-6 levels and NFκB activity. This effect was notably initiated through increased activation of protein phosphatases and increased synthesis of epoxyeicosatrienoic acid (Rompe et al., 2010).

## CONCLUSION

Since its identification in the early 90s, the AT_2_ receptor has been and still is shrouded by controversy, its low expression in the adult and its atypical signaling pathways adding to the challenge of studying this receptor. Thanks to the major advances achieved in the past few years, several studies have confirmed that stimulation of the AT_2_ receptor activates multiple signaling pathways which are linked to beneficial effects on neuronal functions (including excitability, differentiation, and regeneration), inflammation, oxidative stress, and cerebral blood flow (**Figure 1**). Several neurodegenerative diseases (including cognitive deficits and dementia) are closely associated with these neuronal and synaptic dysfunctions (Iadecola, 2004; Zlokovic, 2005; LaFerla et al., 2007; Boissonneault et al., 2009; Mucke, 2009; Nelson et al., 2009). Moreover, an increasing number of studies suggest that the protective effects of ARBs on brain damage and cognition may result not only from the inhibition of AT_1_ receptor effects, but also from the beneficial effect due to unopposed activation of the AT_2_ receptor. Thus, if further research confirms the promising early results obtained with the recently developed selective non-peptide AT_2_ receptor agonist C21/M024, the latter may represent a new pharmacological tool in the fight against neurological cognitive disorders. In addition, unraveling the underlying effects of the AT_2_ receptor on neuronal plasticity may lead to the development of even more potent and selective therapies.

## Conflict of Interest Statement

The authors declare that the research was conducted in the absence of any commercial or financial relationships that could be construed as a potential conflict of interest.
